# Effects of Halophyte Root Exudates and Their Components on Chemotaxis, Biofilm Formation and Colonization of the Halophilic Bacterium *Halomonas Anticariensis* FP35^T^

**DOI:** 10.3390/microorganisms8040575

**Published:** 2020-04-16

**Authors:** Inmaculada Sampedro, Daniel Pérez-Mendoza, Laura Toral, Esther Palacios, César Arriagada, Inmaculada Llamas

**Affiliations:** 1 Department of Microbiology, Pharmacy Faculty, Campus de Cartuja s/n, 18071 Granada, Spain; dpmendoza@eez.csic.es (D.P.-M.); esthepalgo@gmail.com (E.P.); illamas@ugr.es (I.L.); 2 Biomedical Research Center (CIBM), Biotechnology Institute, Avda del Conocimiento s/n, 18100 Armilla, Granada, Spain; 3 Department of Soil Microbiology and Symbiotic Systems, Estación Experimental del Zaidín, Spanish National Research Council (CSIC), Profesor Albareda 1, 18008 Granada, Spain; 4 Xtrem Biotech S.L., European Business Innovation Center, Avenida de la Innovación, 1, 18016 Armilla, Granada, Spain; lauratn28@gmail.com; 5 Department of Forestry Science, Bioremediation Laboratory, Faculty of Agricultural and Forestry Science, University of La Frontera, 01145 Temuco, Chile; cesar.arriagada@ufrontera.cl

**Keywords:** chemotaxis, PGPBs, halophilic bacteria, *Salicornia*, biofilm, root exudates, oleanolic acid

## Abstract

Increase in soil salinity poses an enormous problem for agriculture and highlights the need for sustainable crop production solutions. Plant growth-promoting bacteria can be used to boost the growth of halophytes in saline soils. *Salicornia* is considered to be a promising salt-accumulating halophyte for capturing large amounts of carbon from the atmosphere. In addition, colonization and chemotaxis could play an important role in *Salicornia*-microbe interactions. In this study, the role of chemotaxis in the colonization of the halophilic siredophore-producing bacteria, *Halomonas anticariensis* FP35^T^, on *Salicornia hispanica* plants was investigated. The chemotactic response of FP35^T^ to *Salicornia* root exudates showed optimum dependence at a salt concentration of 5 % NaCl (w/v). Oleanolic acid, the predominant compound in the exudates detected by HPLC and identified by UPLC-HRMS Q-TOF, acts as a chemoattractant. In vitro experiments demonstrated the enhanced positive effects of wild-type *H. anticariensis* strain FP35^T^ on root length, shoot length, germination and the vigour index of *S. hispanica.* Furthermore, these positive effects partially depend on an active chemotaxis system, as the chemotaxis mutant *H. anticariensis* FP35 ΔcheA showed reduced plant growth promotion for all the parameters tested. Overall, our results suggest that chemotaxis responses to root exudates play an important role in interactions between *Salicornia* and halophilic bacteria, enhance their colonization and boost plant growth promotion. Preliminary results also indicate that root exudates have a positive impact on *H. anticariensis* FP35^T^ biofilm formation under saline conditions, an effect which totally depends on the presence of the *cheA* gene.

## 1. Introduction

Increase in the salinization of arable soils is commonly found in semi-arid and arid areas. According to global change scenarios, agriculture under saline conditions has to be regarded as an alternative strategy for improving food production. The principal strategies used to improve crop yields in saline soils involve the use of halotolerant plant growth promoting rhizobacteria (PGPR) and/or halophytes [1]. Halophytes play an important role in saline soil reclamation [2] and phytoremediation of contaminated saline soils [3]. The genus *Salicornia* (*Chenopodiaceae*) is composed of approximately 25–30 plant species which are extremely salt tolerant and widely dispersed in Eurasia, North America and South Africa [4,5]. *Salicornia* species have been the subject of considerable research, particularly in relation to their use in food production [6], their medicinal applications [7,8], their use as a forage crop [9] and their salt tolerance mechanisms [10]. *Salicornia* is also considered to be a promising salt-accumulating halophyte for capturing large amounts of carbon in the atmosphere [11]. 

While there have been few studies of PGPR bacteria associated with this halophyte [12,13], halophilic bacteria of the genus *Halomonas* have been reported to colonize *Salicornia* roots [14]. *H. anticariensis* FP35^T^, an important member of this genus, is a moderately halophilic siderophore-producing bacterium which was isolated from a saline wetland in Malaga, Spain [15] and was characterized in order to produce an exopolysaccharide containing quorum-sensing signalling molecules with biotechnological applications [16,17]. 

Colonization is a major feature of plant-PGPR interactions, while the chemotaxis-mediated responses of PGPR to the root system are required before root colonization can take place [18]. Chemotaxis is based on the concerted action of excitatory and adaptive mechanisms by the sensing of molecular species via membrane chemoreceptors or MCPs (Methyl-Accepting Chemotaxis Proteins) (a.k.a., chemoreceptor sensor proteins) that transduce the signal into an appropriate motor response by the two-component system CheA/CheY [19]. In this pathway, CheA is the central kinase and it has been shown for other bacteria like *Listeria monocytogenes* or *Helicobacter pylori* that its inactivation abolishes all chemotaxis responses [20,21]. Chemotaxis to a plant-derived compound, which facilitates the movement of bacteria and their entry through the plant’s openings, plays an important role in plant colonisation [22]. The responses of the plant growth-promoting rhizobacteria *Azotobacter chroococcum* and *Pseudomonas fluorescens* to roots and their exudates are also involved in interactions between bacteria and vesicular-arbuscular mycorrhizal roots [18]. Ling et al. [23] have reported that organic acids in watermelon root exudates recruit *Paenibacillus polymyxa* SQR-21, while Rudrappa et al. have described how malic acid in plant roots chemically attracts *Bacillus subtilis* FB17 [24]. However, little is known about the responses of halophilic bacteria to chemical and natural compounds or their chemotaxis machinery, with only a few recent studies describing the chemotaxis-mediated responses of *H. anticariensis* FP35^T^ to environmental pollutants [25] and analysing the genome of *Halomonas* strains to search for chemosensory genes and the chemosensory pathways within the genus *Halomonas* [26,27]. 

On the other hand, the formation of biofilms by beneficial bacteria on plant roots, which is indicative of effective plant colonization [28], protects the host plant against external stresses such as pathogen attacks and salinity [29,30]. Exopolysaccharides (EPSs) secreted by halophilic bacteria of the genus *Halomonas*, including those produced by *H. anticariensis* FP35^T^, have been reported to be involved in biofilm formation [31,32]. To understand the interactions between halophytes and FP35^T^, it is crucial to study mechanisms such as chemotaxis and biofilm formation in order to develop strategies for optimizing plant colonization.

This study aims to analyse the effects of *Salicornia* root exudates on the chemotactic responses and biofilm formation of the halophilic bacterium *H. anticariensis* FP35^T^ and to evaluate their impact on halophyte colonization. We also provide information on halophyte growth conditions that enhance the recruitment of halophilic bacteria in the rhizosphere and boost *Salicornia* growth which could be effective in combating soil salinity, water scarcity and global climate change behaviour.

## 2. Materials and Methods 

### 2.1. Bacterial Strains and Culture Conditions

Wild-type *Halomonas anticariensis* strain FP35^T^ [15], as well as the mutant strains FP35 ΔcheA [25] and FP35 ΔcheA pJN105-cheA (this study), were cultured in MY liquid medium, composed of 3 g malt extract, 3 g yeast extract, 10 g glucose and 5 g peptone per litre [33,34], modified with a balanced mixture of sea salt solution [35] up to a final concentration of 2, 5, 7.5 and 12.5% (w/v) at 32 °C. *H. salina* F8-11^T^ [36], *H. elongata* 1H9^T^ [37], *Pseudomonas halophila* CECT 5286^T^ [38], *H. ventosae* Al-12^T^ [39], *H. rifensis* HK31^T^ [40], *H. almeriensis* M8^T^ [41], *H. cerina* SP4^T^ [42], *H. eurihalina* FP6^T^ [43], *H. stenophila* N12^T^ [44], *H. ramblicola* RS16 [45] and *H. organivorans* G16.1^T^ [46] were cultured at 32 °C in MY liquid medium with 5% (w/v) sea salt solution. The antibiotics rifampicin (50 µg mL^−1^), gentamycin (20 µg mL^−1^) and kanamycin (50 µg mL^−1^) were selectively used to grow the mutant strains. 

### 2.2. Swimming Motility Assay

The swimming motility of the bacterial strains was tested using a plate-based assay described by Ha et al. [47]. Briefly, overnight cultures of bacterial strains were diluted to OD_600_ of 0.2 and, using toothpick bacteria, were point-inoculated onto M8 plates containing 0.3% agar. Motility was observed after 16 h of incubation at 32 °C. 

### 2.3. Identification of Chemosensory Systems in H. anticariensis FP35^T^

The draft genome sequence of *H. anticariensis* FP35^T^ was analysed using the RAST Annotation Server [48] and the Integrated Microbial Genomes Database [49]. All the genes found in the *H. anticariensis* FP35^T^ genome that coded putative chemosensory related proteins were compared to the well described *E. coli* chemotaxis proteins (CheA, CheW, CheR, CheB, CheY, CheZ and MCPs) using NCBI available sequences.

### 2.4. Cloning of CheA ORF in a Multicopy Plasmid

The chromosomal *cheA* gene of *H. anticariensis* FP35^T^ was disrupted in a single step by Campbell-type integration of the entire plasmid by homologous recombination using the suicide plasmid pVIK112 as previously described by our research group [25]. For the complementation analysis of mutant FP35 ΔcheA, molecular biology experiments were performed according to standard protocols and the manufacturer’s instructions. A DNA fragment flanking a *cheA* region of the 1922 bp gene was amplified from total DNA previously extracted from *H. anticariensis* FP35^T^ using the primers mcheA-EcoRI-Fw3 (5′-GGAAAGAATTCGGATGAAGCCTCCTATTCG-3′) and mcheA-XbaI-Rev2 (5′-GCGTCTAGACTAGAACTCACTCCAGTCGTC-3′), cloned into pCR-XL-TOPO (Thermo Fisher Scientific, Waltham, MA, USA) and then sequenced. A correct EcoRI/XbaI insert was isolated and subcloned into the multicopy plasmid pJN105 [50] which was previously digested with the same restriction enzymes. OmniMax *E. coli* electro-competent cells (Thermo Fisher Scientific, Waltham, MA, USA) were transformed by electroporation according to the manufacturer’s instructions, and the transformants were selected in LB agar plates supplemented with gentamicin (20 µg mL^−1^). A correct pJN105-*cheA* clone and its corresponding empty vector control pJN105 were transferred by biparental mating to the mutant strain *H. anticariensis* FP35 ΔcheA using the *E. coli* β2163 as donor strain [51] as described elsewhere [52].

### 2.5. Extraction of Root Exudates from Salicornia Plants

Briefly, six-month-old plant roots were rinsed thoroughly with deionised water and were then placed in a black plastic wrapped beaker containing 200 mL Milli-Q water at 25–20 °C (day-night) during a 10–14 h light-dark period of illumination. After 24 h, the solution containing the released root exudates in the beaker was filtered through a 0.22 µm membrane and then stored at −20 °C [53]. 

### 2.6. Analysis of Salicornia Root Exudates Using UPL-HRMS Q-TOF

The root exudates were collected, freeze-dried, dissolved in 10% (w/v) methanol and analyzed using an ultra high-pressure liquid chromatograph (UPLC) (Acquity UPLC^®^, Waters Corporation, Milford, MA, USA) coupled to a SYNAPT G2 Q-TOF high resolution mass spectrometer (HRMS; Waters Corporation, Milford, MA, USA). Mass spectrometry was carried out in positive ionization electrospray mode (ESI+). The data obtained were processed by MassLynx^™^ software (Waters Corp., Milford, MA, USA). The growth of *H. anticariensis* FP35^T^ in presence or absence of the main component of root exudates was tested using the assay previously described in Section 2.2.

### 2.7. Chemotaxis Assays 

#### 2.7.1. Qualitative Capillary Chemotaxis Assay 

The qualitative capillary chemotaxis assay was carried out as described elsewhere [54]. Briefly, the washed cells were suspended in chemotaxis buffer (CB) to reach a final optical density of 0.1 (OD_600_) and then placed in a chamber containing a U-shaped tube, microscope slide and cover slip. Microcapillary tubes (1 µL) sealed at one end were filled with the *Salicornia* root exudates extracted as previously described in Section 2.5 and 2% (w/v) low-melting temperature agarose and then inserted into the pool of bacterial cells. Negative controls (CB) were included in all experiments. The chemotactic response at the mouth of the capillary tube was visualized at 0 and 5 min using dark-field microscopy (Olympus IX73) with an Olympus TH4-100 halogen illuminator and was photographed using an Olympus DP73 CCD camera (version 1.8. software). Dark-field illumination was generated using a condenser with a contrast phase ring (Ph2) at an NA of 0.55 with an UPlanFLN 4x NA 0.13 objective. The images were processed by adding contrast and brightness using Adobe Photoshop Lightroom software. The data were normalized to time 0 using the Matlab R2013a program to obtain a heat map of bacterial chemotactic responses. 

#### 2.7.2. Quantitative Capillary Assay 

A modified version of the capillary assay described by Adler [55] was used to quantify the chemotactic response of bacteria to a chemical gradient. Exponentially grown cells were harvested by centrifugation at 13,000 rpm for 5 min, washed and suspended in chemotaxis buffer (CB) containing 40 mM K_2_HPO_4_/KH_2_PO_4_, 0.05% (w/v) glycerol, 10 mM EDTA, pH 7.0 at 2, 5, 7.5 and 12.5% (w/v) sea salt solution to reach a final optical density of 0.1 (OD_600_). Microcapillary tubes (1 µL) sealed at one end were filled with the *Salicornia* exudates extracted as previously described in Section 2.5 or high purity commercially available oleanolic acid from Sigma Aldrich and immersed in the cell suspension for 1 h. The tubes were then removed and their exterior was rinsed with sterile water. The contents of each tube were transferred to tubes containing CB by centrifugation (13,000 rpm), diluted (10^−3^ and 10^−4^) and then spread on appropriate MY agar plates to quantify the chemotactic response (CFU per capillary tube). Negative controls were performed in all experiments. The results were expressed as the increase in CFU for each compound tested and were normalized using CB. Data were expressed as the mean ± SEM of at least three independent experiments, each of which had three technical replicates.

### 2.8. Biofilm Formation Assay 

The biofilm formation of *H. anticariensis* FP35^T^ and the mutant FP35 ΔcheA in the presence of root exudates was measured as described elsewhere [56]. Briefly, overnight cultures of each bacterium were grown at 32 °C up to OD_600_ 1.0. The cells were then diluted 1/100, and 96-well microtiter plates were filled with 100 µL of the dilution. Root exudates (10% and 25% (v/v)) or oleanolic acid (0.1 and 1 mM) were added to the medium in each well. Each treatment was replicated four times. After static incubation at 32 °C for 48 h, the biomass of biofilm formed by each bacterium was determined by a crystal violet assay at 0.1% (w/v) according to O´Toole et al. [56]. Biofilm formation was quantified by measuring the OD_590_ for each well using a Tecan Sunrise microplate reader and XFluor V4.51 software. 

### 2.9. Salicornia Seed Bacterization and Germination Assay 

*Salicornia* seeds (provided by Alsa Garden, Niederhaslach, France) were surface-disinfected with 10% w/v NaClO solution for 10 min and with 70% v/v ethanol for a further 5 min. They were then washed three times with sterile distilled water. To check the efficiency of the sterilisation process, the seeds were placed on plates containing MY medium and incubated for 4 days. The seeds were bacterized according to the method described by Jha et al. with slight modifications [57]. Overnight cultures of *H. anticariensis* FP35^T^ and *H. anticariensis* FP35 ΔcheA were centrifuged at 10,000 rpm for 5 min, and each pellet was washed with PBS (pH 7.2). The pellets were re-suspended in PBS to reach a final optical density of 0.6 (OD_600_). For bacterization, the sterilised seeds were immersed for 24 h in bacterial suspension containing 0.1% (w/v) carboxymethyl cellulose. As a control, seeds were suspended in PBS containing 0.1% (w/v) carboxymethyl cellulose without bacterial culture. Germination tests were performed on 30 bacterized seeds in sterilised petri dishes moistened with NaCl solution (0 and 0.25 mol L^−1^). All treatments were incubated at 27 ± 0.5 °C and 35 ± 1% humidity and carried out in triplicate. The final percentage of germination was determined after 30 days by measuring root and shoot length. The vigour index was calculated according to the formula described by Abdul Baki and Anderson [58]: vigour index = mean root length + mean shoot length × germination (%).

In order to quantify root bacterial colonization, one-month-old plant roots were rinsed thoroughly with deionised water, and 0.1 g of mixed root tips was ground with 1 mL sterilized distilled water using a mortar. The suspensions were diluted, plated on MY medium using 5% sea salt solution (w/v) and then counted after incubating the plates for 2 days at 32 °C.

### 2.10. Statistical Analyses

The data obtained were subjected to ANOVA analysis, and multiple pair-wise comparisons were made using the Tukey test.

## 3. Results

### 3.1. Assessment of the Chemotactic Response of H. anticariensis FP35^T^ to Salicornia Root Exudates

Bacterial motility was assayed in order to select halophilic motile bacteria for further chemotaxis study. Twelve halophilic bacteria: *Halomonas anticariensis* FP35^T^, *H. rifensis* HK31^T^, *H. elongata* 1H9^T^, *H. almeriensis* M8^T^, *H. cerina* SP4^T^, *H. eurihalina* FP6^T^, *H. ventosae* Al-12^T^, *H. stenophila* N12^T^, *H. ramblicola* RS16^T^, *H. salina* F8-11^T^, *H. organivorans* G16.1^T^ and *Pseudomonas halophila* DSM 3051 were tested for motility. Seven of the bacteria tested showed some degree of swimming motility, and *H. anticariensis* FP35^T^, which had the highest motility, was selected (data not shown). 

**Figure 1:**
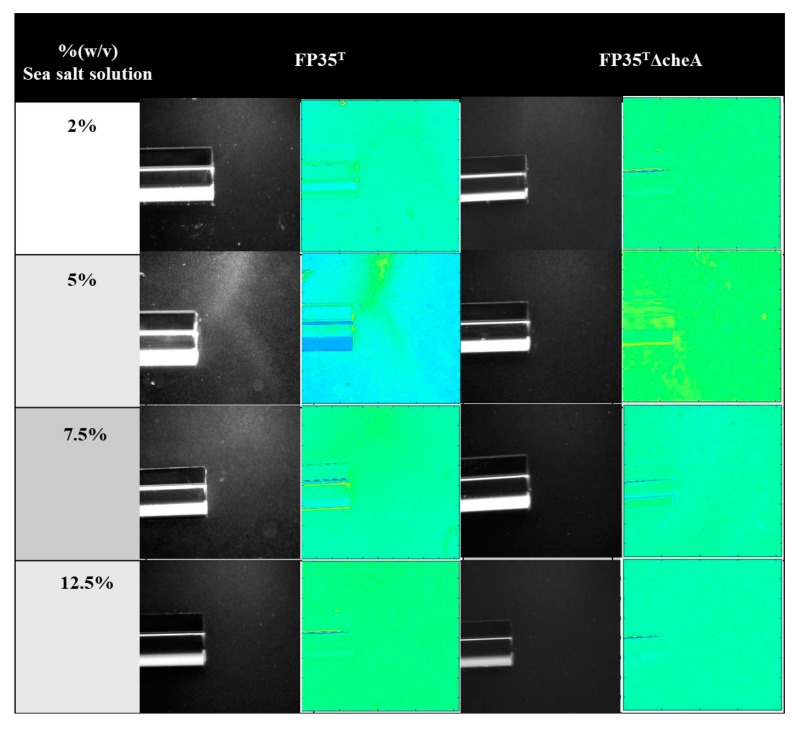
Qualitative capillary chemotaxis assays comparing responses of wild-type *H. anticariensis* FP35^T^ and the mutant strain FP35 ΔcheA to root exudates grown at different sea salt solution concentrations. All photographs were taken after 10 min. First and third columns: dark-field images of cells gathered at the mouth of capillary tubes containing attractants; second and fourth columns: heat map of normalized images (Matlab R2013a).

In this study, we have analyzed the draft genome sequence of FP35^T^ and we have found one cluster of chemotaxis-related genes and 21 chemoreceptors. The gene cluster seems to govern the chemotactic behavior of FP35^T^ and it is very similar to the cluster from *Escherichia coli* (Figure S1). To investigate the chemotactic response of the moderately halophilic bacterium *H. anticariensis* to *Salicornia* root exudates, chemotaxis experiments were performed using the wild-type strain and the mutant FP35 ΔcheA. The *cheA* gene of FP35^T^ coding for MCPs, which plays an important role in the transduction module of the chemotaxis pathway due to its homology with *E. coli,* was disrupted in a previous study [25]. The chemotactic response of *H. anticariensis* FP35^T^ and its mutant FP35 ΔcheA to *Salicornia* root exudates was first qualitatively analyzed in salt concentrations ranging from 2 to 12.5% (w/v) in order to determine the optimal saline concentration for bacterial chemotaxis. Of the different sea salt solution concentrations tested, the results indicate that the optimal concentration is 5% (w/v) for the chemo-attraction of wild-type cells to root exudates (Figure 1). As expected, the FP35 ΔcheA mutant was observed to lack a chemotaxis response under all the experimental conditions analysed. Figure S2 shows the absence of a detectable chemotaxis response of FP35^T^ chemotaxis buffer (negative control) at the optimal 5% (w/v) concentration for bacterial chemoattraction.

**Figure 2:**
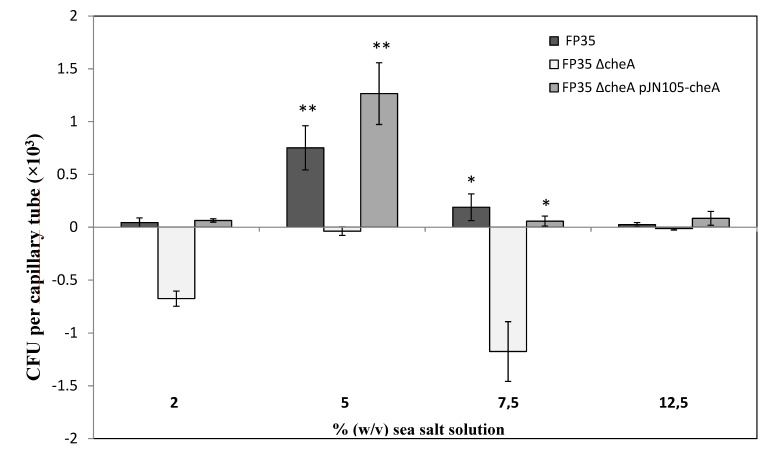
Quantitative capillary chemotaxis assays comparing responses of wild-type *H. anticariensis* FP35^T^ and mutant strains FP35 ΔcheA and FP35 ΔcheA pJN105-cheA to root exudates grown at different % (w/v) sea salt solution concentrations. The results represent the mean of three independent biological samples performed in triplicate, with error bars indicating standard errors. The graph shows the increase in CFU in capillaries normalized by the negative control (CB). The negative CFU values in the capillaries reflect negative chemotaxis. Comparison of different strains tested using the Tukey test (* *p* ≤ 0.05; ** *p* < 0.01).

To confirm the role of *cheA* observed in chemotactic qualitative experiments, quantitative assays were carried out in the same range of salt concentrations (Figure 2). Of the different sea salt solution concentrations tested, the results confirm that the optimal concentration is 5% (w/v) for the accumulation of wild-type cells in capillary tubes containing root exudates (Figure 2). By contrast, the FP35 ΔcheA mutant showed an accumulation of below or close to zero under all the experimental conditions analysed. The negative CFU values at concentrations of 2 and 7.5% (w/v) reflect negative chemotaxis. The *cheA* required for positive chemotaxis was confirmed by the complementation of the FP35 ΔcheA mutant with a copy of the *cheA* gene *in trans* (pJN105-*cheA*; Figure 2).

### 3.2. Chemoattraction to Salicornia Root Exudates in a Variety of Halophilic Bacteria

**Figure 3:**
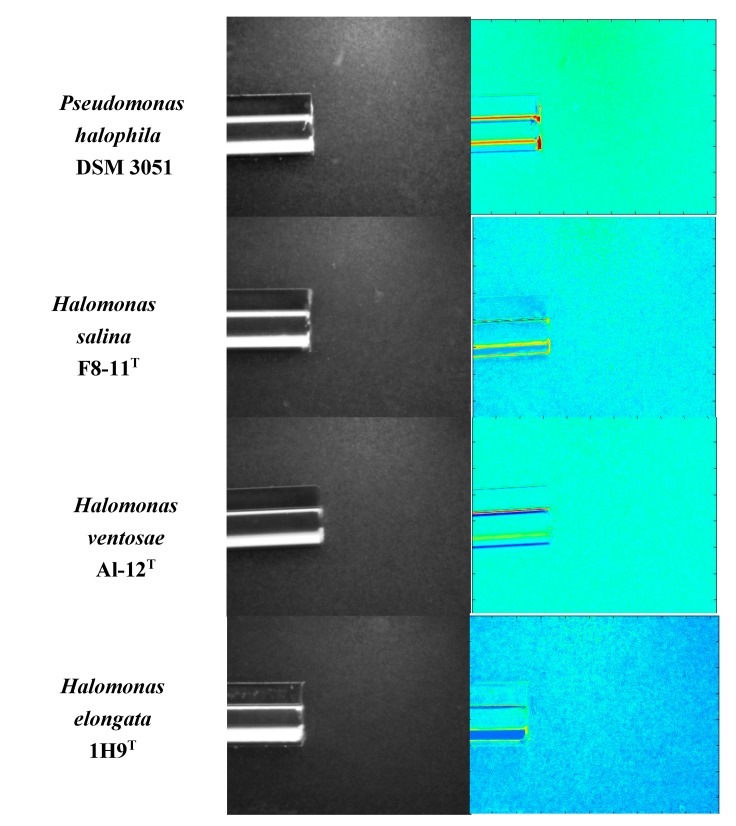
Qualitative capillary chemotaxis assays comparing responses of four halophilic bacteria to root exudates grown at a 5% (w/v) concentration of sea salt solution. All photographs were taken after 10 min. First column: dark-field images of cells gathered at the mouth of capillaries containing exudates; second column: heat map of normalized images (Matlab R2013a).

After confirming the positive effect of *Salicornia* root exudates on the chemotactic response of *H. anticariensis* FP35^T^, we hypothesized that these root exudates could also act as chemoattractants to other halophilic bacteria. A qualitative assessment of swimming motility showed an increase in halo size in the other four bacteria, *H. ventosae* Al-12^T^, *H. elongata* 1H9^T^*, H. salina* F8-11^T^ and *Pseudomonas salina* DSM 3051, all grown at an optimal 5% (w/v) salt concentration previously observed for *H. anticariensis* FP35^T^. Only two of these bacteria, *P. halophila* DSM 3051 and *H. salina* F8-11^T^, exhibited chemoattraction to *Salicornia* root exudates (Figure 3). Nevertheless, *Salicornia* exudates elicited a lower chemotaxis response to these two strains as compared to that to *H. anticariensis* FP35^T^. 

### 3.3. Identification of Salicornia Exudate Composition Using UPLC - HRMS Q-TOF 

**Figure 4:**
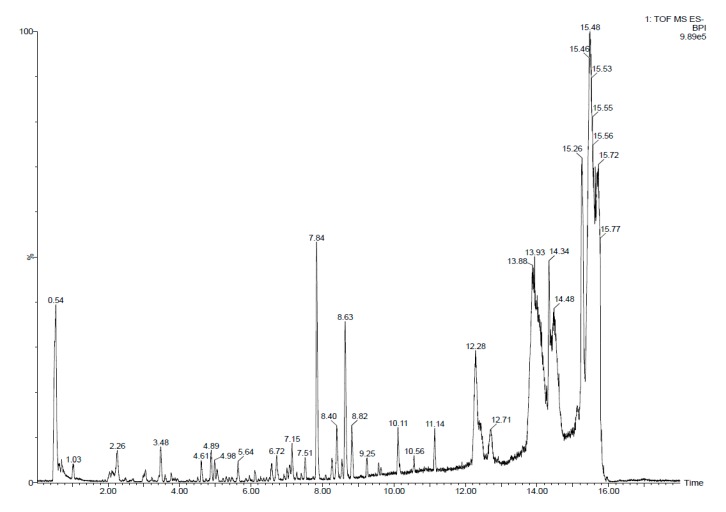
TIC spectrum obtained from *Salicornia* exudates.

**Figure 5:**
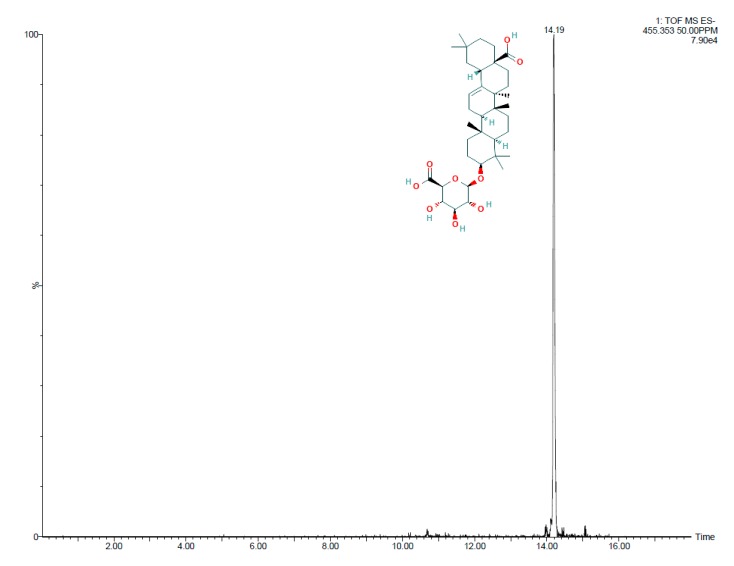
UPLC-HRMS Q-TOF spectra obtained from *Salicornia* exudates.

Electrospray quadrupole time-of-flight mass spectrometry (Q-TOFS MS) was used to identify metabolites produced by the exudates tested. Figure 4 shows the total ion chromatogram (TIC) spectrum of the *Salicornia* root exudates. Profiles of the root exudates from *Salicornia* plants demonstrate the presence of different peaks, the most abundant of which according to area was detected at a retention time of 14.16 min. This peak, corresponding to [M + H]^+^ at m/z 455.3525, afforded the molecular formula C_30_H_48_O_3_ and was identified by LC-HRMS to correspond to oleanolic acid (Figure 5).

The concentration of this compound in the *Salicornia* root exudates sampled was 90 µM. An optimum growth of the bacterium FP35^T^ in presence of oleanolic acid (at concentration of 0.1 mM) was observed (Figure S3). The bacterium growth pattern after 14 h revealed similar values in absence or in presence of this compound (OD_600_ control: 1.006 ± 0.09 vs. OD_600_ oleanolic acid, 0.1 mM: 1.16 ± 0.14).

### 3.4. Assessment of Chemotactic Response of H. anticariensis FP35^T^ to Oleanolic Acid

We evaluated the chemotactic responses of FP35^T^ to the predominant compound, oleanolic acid (Sigma-Aldrich, Missouri, USA), in *Salicornia* exudates. We carried out experiments at a 5% (w/v) concentration of a sea salt solution which generates higher background accumulation of wild-type cells in capillaries containing root exudates. The concentrations of oleanolic acid (0.1 and 1 mM) tested in this study were selected according to the positive chemotaxis response previously observed for *Pseudomonas* and *Bacillus* to other compounds detected in root exudates in this range of concentrations [18,59].

**Figure 6:**
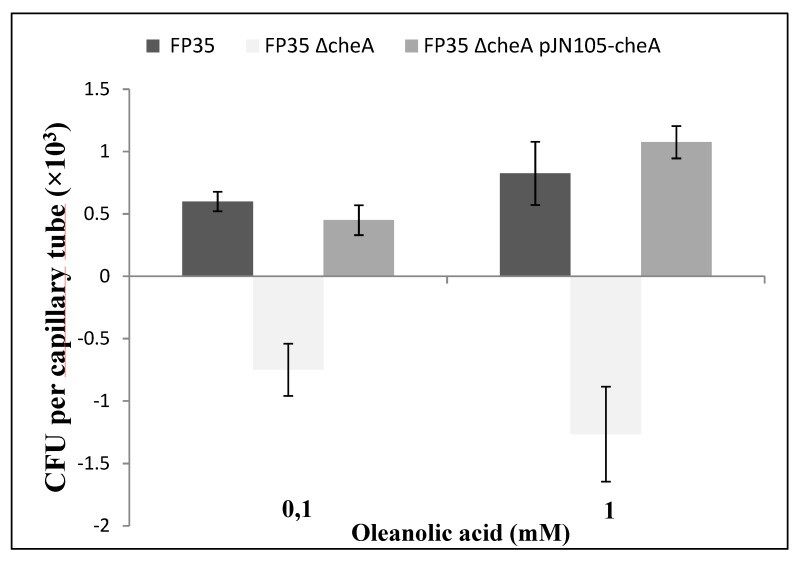
Quantitative capillary chemotaxis assays comparing responses of wild-type *H. anticariensis* FP35^T^ and its mutant strains FP35 ΔcheA and FP35 ΔcheA pJN105-cheA, grown in 5% (w/v) sea salt solution, to oleanolic acid. The results represent the mean of three independent biological samples performed in triplicate, with error bars indicating standard errors. The graph shows the increase in CFU in capillaries normalized by the negative control (CB). The negative values for CFUs in the capillaries reflect negative chemotaxis.

The wild-type strain FP35^T^ showed positive chemotaxis for oleanolic acid tested at concentrations of 0.1 mM and 1 mM (Figure 6), while the FP35 ΔcheA mutant showed negative CFU values, corresponding to negative chemotaxis. As expected, positive responses to oleanolic acid were restored in the complemented mutant FP35 ΔcheA pJN105-cheA (Figure 6). Background accumulation of cells, at close to 1 × 10^3^, found in capillaries containing 1mM of oleanolic acid was similar to that in the capillaries containing *Salicornia* exudates. Negative chemotaxis response was observed for ethanol, the solvent used to dissolve oleanolic acid (data not shown).

### 3.5. Evaluation of H. anticariensis FP35^T^ Biofilm Formation in Response to Salicornia Root Exudates and Oleanolic Acid 

**Figure 7:**
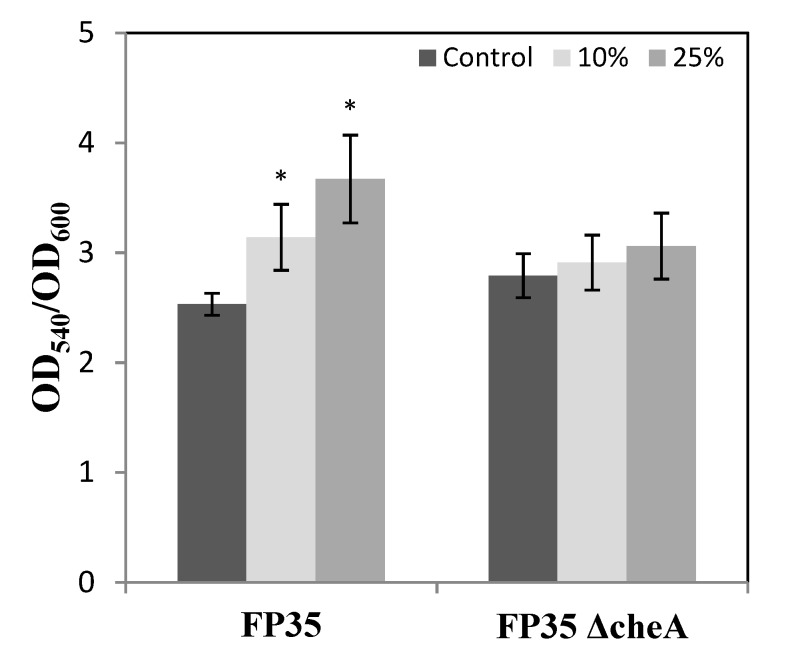
Biofilm formation of wild-type *H. anticariensis* strain FP35^T^ and the FP35 ΔcheA mutant grown in 5% (w/v) sea salt solution induced by 10% and 25% (w/w) root exudate concentrations of *Salicornia*. The results represent the mean of three independent biological samples performed in quadruplicate, with error bars indicating standard deviations. Comparisons with respect to the control for each strain tested were made using the Tukey test (* *p* ≤ 0.05).

To ascertain their impact on the biofilm formation of *H. anticariensis* FP35^T^, 10 and 25% (v/v) concentrations of *Salicornia* root exudates were added to the cultures grown in 5% (w/v) sea salt solution. The results indicate that both these concentrations of root exudates significantly increased the biofilm biomass of FP35^T^ (Figure 7). However, no statistically significant differences were observed in the chemotaxis mutant FP35 ΔcheA with respect to the two exudate root concentrations tested. This suggests that an active chemotaxis system is required in FP35^T^ order to promote biofilm formation by *Salicornia* root exudates. Because oleanolic acid is the predominant compound in *Salicornia* exudates, its impact in the biofilm formation of FP35^T^ was also evaluated. Changes in biofilm in this case were not observed because no significant differences were found between the biofilm formation in the presence of this compound and in the presence of ethanol (the compound used to dissolve this synthetic compound with limited solubility) (OD_540_/OD_600_ control ethanol, 0.1 mM or 1 mM: 1.29 ± 0.65; 1.23 ± 0.10 vs. OD_540_/OD_600_ oleanolic acid, 0.1 mM or 1 mM: 0.57 ± 0.14; 1.11 ± 0.44).

### 3.6. Salicornia Plant Reactions to Inoculation with H. anticariensis strain FP35^T^ and its Mutant Strain FP35ΔcheA

The effect of the wild-type *H. anticariensis* strain FP35^T^ and its mutant *H. anticariensis* strain FP35 ΔcheA on the root length, shoot length, germination and vigour index of *Salicornia* was tested. An increase in the shoot and root length of *Salicornia* seeds was observed following inoculation with *H. anticariensis* strain FP35^T^ as compared to the non-inoculated control (Table 1). The bacterization of seeds by the halophilic bacteria also significantly increased the percentage of germination. In stark contrast, seeds bacterized by *H. anticariensis* strain FP35 ΔcheA only boosted root growth, while shoot length and the percentage of germination were similar to those for the non-inoculated control (Table 1). Furthermore, the 153% increase in the vigour index caused by inoculation with the wild-type strain exceeded the 79% increase in that for the chemotaxis mutant. These results highlight the important role of chemotaxis in the PGPR activity of *H. anticariensis* strain FP35^T^ in *Salicornia*. Root colonization of the bacterial strains under seed bacterization conditions was tested, which showed a 2.8-fold increase in the *H. anticariensis* FP35^T^ colonization of *Salicornia* roots (6.3 × 10^5^ ± 0.42) CFUs g^−1^ dry root) as compared to colonization by the chemotaxis mutant (2.3 × 10^5^ ± 0.29) CFUs g^−1^ dry root).

## 4. Discussion

Increase in soil salinity, which constitutes an enormous challenge for agriculture, needs to be tackled in order to come up with solutions to achieve sustainable crop production. One of the principal strategies for improving crop yields in saline soils is the use of PGPR bacteria for the promotion of halophyte growth in this type of soil. Rhizosphere colonization by PGPR bacteria is associated with root exudates [60] which play an important role in the rhizosphere and act as signals that mediate root-microorganism interactions [61]. Chemotaxis is a key player in initiating crosstalk between plant roots and PGPR bacteria [62]. Though extensively studied in model organisms such as *Escherichia coli* and *Pseudomonas* spp. [19], little is known about these chemotactic bacterial systems in halophilic bacteria such as those of the genus *Halomonas* [25,26,27], which have, however, been studied in relation to *Salicornia* root colonization [63]. We assayed the motility of bacteria belonging to the genus *Halomonas* that were isolated from the halophyte rhizosphere. *H. anticariensis* strain FP35^T^, which had the highest motility of all the bacteria tested, was selected. 

The halophilic bacterium *H. anticariensis* FP35^T^ has plant growth-promoting properties such as siderophore production, one of the principal advantages of this type of bacterium, which excludes other microorganisms and reduces competition for constituents of root exudates [64]. Strain FP35^T^ produces 2,3-butanediol during glucose fermentation, as well as nitrogenase and nitrate and nitrite reductase, and hydrolyses gelatine [15]. FP35^T^ also produces sulphated exopolysaccharides with many biotechnological applications [17], as well as quorum-sensing signalling molecules [16].

In a previous study, an identification of the draft genome sequence of *H. anticariensis* FP35^T^ was performed [65]. Analysing this genome sequence, we have found one cluster of chemotaxis-related genes and twenty-one chemoreceptors. This gene cluster seems to govern the chemotactic behavior of FP35^T^ and it is very similar to the cluster from *Escherichia coli* including the *cheA* gene. Similar results were previously described for this strain and other species from the genus *Halomonas* [27]. Given its importance in the chemotaxis pathway of other bacteria, the *cheA* gene, through its disruption in strain FP35^T^, was found to play a crucial role in chemotactic responses to environmental hydrocarbon pollutants [25]. We carried out chemotaxis and biofilm formation experiments to investigate how *Salicornia* root exudates mediate the enhanced root colonization of *H. anticariensis* strain FP35^T^. The chemotactic response of the halophilic bacterium FP35^T^ to *Salicornia* root exudates was analysed using qualitative and quantitative chemotaxis experiments. In addition, a complemented chemotaxis mutant strain FP35 ΔcheA pJN105-cheA was constructed in order to corroborate the chemotaxis results. *Salicornia* root exudates were observed to act as chemoattractants for strain FP35^T^ grown in concentrations of sea salt solution ranging from 2 to 12.5% (w/v), with the strongest chemotactic response found at 5% (w/v). These results were confirmed by the *cheA* mutant’s lack of chemotaxis response and the chemoattraction of the complemented mutant strain FP35ΔcheA pJN105-cheA. Interestingly, in a previous study, strain FP35^T^ showed the strongest chemoattraction to environmental pollutant compounds such as phenol and naphthalene when grown in a sea salt solution concentration of 7.5% (w/v) [25]. These findings point to the significance of salt concentrations in the chemotaxis system and of the chemotaxis responses of *H. anticariensis* FP35^T^ to different substances found in the soil. To the best of our knowledge, only few studies analyse the chemotactic behaviour of *Halomonas* strains using swimming assays with times of incubation higher than 24 h [27,66].

The chemotaxis response of FP35^T^ to root exudates found differed from that observed in this study for other species of the genus *Halomonas* spp. This indicates that the genus *Halomonas* spp. is comprised of bacteria with chemosensory signalling mechanisms of varying complexity, a conclusion previously reached in relation to species of the genus *Pseudomonas* spp. For example, although tetrachloroethylene acts as a chemoattractant for *P. putida* F1 and *P. stutzeri* OX1 [67,68], it has been found to be a chemorepellent for *P. aeruginosa* PAO1 [69,70].

Chemoattraction and biofilm formation by root exudates are associated with the colonization process [71]. *Salicornia* exudates act as chemoattractants for FP35^T^ and as stimulators of biofilm formation at sea salt concentrations of 5% (w/v), the optimum concentration for chemoattraction. Previous studies have indicated that tomato and *Arabidopsis* root exudates increase biofilm formation in different species of the genus *Bacillus* [24,72]. Furthermore, a variety of exudate components such as citric acid, malic acid and fumaric acid have been reported to stimulate the biofilm formation of bacteria [59,71]. However, to our knowledge, little is known about the impact of halophyte exudates on *Halomonas* biofilm. As previous studies of other bacteria of the genus *Bacillus* have suggested [59,72], exudates could act as a carbon source and the growth of FP35^T^ on *Salicornia* exudates may alter the metabolism of this bacterium in a manner favourable to biofilm formation. 

On the other hand, we characterized *Salicornia* exudates in order to determine which constituent is related to FP35^T^ chemoattraction. Plant root exudates are highly complex compound mixtures and vary according to the plant development stage and environmental conditions [73], although some of the constituents of these compound mixtures are unique to certain plants species [74]. Profiles of *Salicornia* exudates showed a high-intensity peak due to oleanolic acid (OA), a pentacyclic triterpenoid compound widely distributed in the plant kingdom. OA has a variety of biological functions in nature, with several promising pharmacological applications [75], plays an important role in regulating the virulence pathway of pathogens such as *Ralstonia solanacearum* [76], appears to be the principal component of *Salicornia* root exudates and acts as a chemoattractant for FP35^T^. The levels of bacterial cell accumulation caused by OA were similar to those caused by chemotaxis to exudates, indicating that this organic acid may be the principal mediator of *Salicornia* plant-bacterium interactions.

Chemotactic reactions to OA were found to be positive at concentrations of 0.1 mM and 1 mM. At these concentrations, a positive chemotaxis response has also been observed to other compounds detected in root exudates such as the gamma-aminobutyric acid (GABA) chemoreceptor in *Pseudomonas putida* strain KT2440 [77], as well as oxalic and malic acids in *Bacillus amyloliquefaciens* SQR9 [59] and *Pseudomonas fluorescens* [18]. An unexpected chemorepellent response of the chemotaxis mutant FP35 ΔcheA to root exudates (at sea salt concentrations of 2 and 7.5%) and OA was observed. Considering that CheA is an essential protein in the chemotaxis signalling cascade in *E. coli* (bacteria model of chemotaxis signal transduction), the *cheA* mutant should be non-chemotactic at all. A previous study indicated that a cytoplasmic chemoreceptor, TlpD, in *Helicobacter pylori* form an autonomous signaling unit and mediate a repellent chemotaxis response to conditions that promote oxidative stress [78]. TlpD is able to localize to the pole and recruits CheW, CheA, and at least two CheV proteins to this location operating independently of other chemoreceptors to organize a chemotaxis signaling complex that mediate this chemorepellent response. The inactivation of *cheA* abolishes chemotaxis responses but could benefit, under certain conditions, the operating action of cytoplasmic chemoreceptors that could change the role of the principal kinases in the chemotaxis system. Future studies should be performed to increase the knowledge of cytoplasmic chemoreceptors in representatives of the genus *Halomonas* that could alter the function and the role of chemoreceptor sensor proteins like CheA.

In this study it was difficult to determine the biofilm formation of FP35^T^ caused by oleanolic acid. The levels of biofilm formation in the presence of this compound were similar to those caused by ethanol (used to dissolve this synthetic compound with limited solubility). It has been previously demonstrated that low concentrations of ethanol stimulate biofilm formation in other bacteria such as *Pseudomonas aeruginosa* [79] and this effect can be a problem when measuring the real biofilm formed by FP35^T^.

The positive relationship between the PGPR *H. anticariensis* strain FP35^T^ and *Salicornia* root exudates could contribute to preferential colonization and stimulation of seed germination. In vitro experiments were carried out to examine the growth-promoting effects of strain FP35^T^ on *Salicornia* seeds under sterile conditions. Seeds bacterized with the halophilic bacterium FP35^T^ showed an increase in germination percentages, as well as shoot and root length, as compared to the non-inoculated control. Similar results were obtained by the inoculation of *Salicornia* seeds with halotolerant bacteria such as *Brachybacterium saurashtrense* and *Pseudomonas* sp. strain JG10 [53]. However, seeds bacterized with the chemotaxis mutant FP35 ΔcheA only showed an increase in root length as compared to the non-inoculated control. Deletion of the gene *mcpG* coding for a GABA chemoreceptor or the gene *cheA3* coding for a histidine kinase in *P. putida* KT2440 reduced root colonization that requires chemotaxis [80,81].

This study indicates that the *cheA* gene coding for MCPs could play an important role in root colonization by FP35^T^. Furthermore, the increase in biofilm formation, previously observed only in the wild-type strain in the presence of *Salicornia* exudates, could enable the bacterium to colonize the roots more effectively. Our results suggest that there is a connection between the absence of chemotaxis and the reduction in the vigour index and colonization of halophyte seeds.

## 5. Conclusions

This study demonstrates that *Salicornia* exudates act as chemoattractants for the halophilic bacterium *H. anticariensis* FP35^T^. Our results also indicate that salt concentration influences chemotactic responses, with optimum levels of chemoattraction observed at a sea salt concentration of 5% (w/v). Oleanolic acid, the principal constituent of *Salicornia* exudates, appears to chemotactically mediate plant-bacterium interactions. The chemotactic responses and increased biofilm formation observed in this study suggest that halophyte exudates have the potential to expand the colonization of these salt-tolerant plants by halophilic bacteria. The enhancement of bacterial recruitment could be a potentially effective strategy for promoting halophyte growth and lead to major benefits for agriculture under saline conditions.

## Figures and Tables

**Table 1:** Effect of wild-type *H. anticariensis* strain FP35^T^ and its mutant *H. anticariensis* FP35 ΔcheA on shoot length, root length, germination and vigour index of *Salicornia*.

Bacterial Inoculation	Shoot (cm)	% Increase	Root (cm)	% Increase	Germination (%)	Vigour Index	% Increase
Control	0.159 ± 0.06a		0.236 ± 0.11a		29	12.52	
*H. anticariensis* FP35^T^	0.179 ± 0.08ab	12,5%	0.679 ± 0.19b	287%	37	31.7	153%
*H. anticariensis* FP35 ΔcheA	0.152 ± 0.05a	0%	0.548 ± 0.13b	232%	32	22.4	79%

Data expressed as mean ± standard deviation. Data in columns with different letters differ significantly according to the Duncan test (*p* < 0.05).

## Supplementary Materials

The following are available online at https://www.mdpi.com/2076-2607/8/4/575/s1, Figure S1: Chemosensory cluster identified in the draft genome sequence of *H. anticariensis* FP35^T^. Figure S2: Qualitative capillary chemotaxis assays comparing responses of wild-type *H. anticariensis* FP35^T^ and the mutant strain FP35 ΔcheA towards chemotaxis buffer grown at a concentration of 5% (w/v) of sea salt solution. Figure S3: *H. anticariensis* FP35^T^ growth in M8 plates containing 0.3% agar in absence or presence of oleanolic acid (A and B, respectively).
