# Supplementary material for: Effects of Halophyte Root Exudates and Their Components on Chemotaxis, Biofilm Formation and Colonization of the Halophilic Bacterium Halomonas Anticariensis FP35T
Source: Microorganisms. 2020 Apr 16;8(4):575. doi: 10.3390/microorganisms8040575 (PMC7232322; doi:10.3390/microorganisms8040575)
Supplement: Supplementary file 1 [file microorganisms-08-00575-s001.pdf]

Effects of halophyte root exudates and their components on chemotaxis, biofilm formation and colonization of the halophilic bacterium *Halomonas anticariensis* FP35^T

Inmaculada Sampedro[1,2*], Daniel Pérez-Mendoza[1,3], Laura Toral[4], Esther Palacios[1,2], César Arriagada[5], Inmaculada Llamas[1,2]

[1]Department of Microbiology, Pharmacy Faculty, Campus de Cartuja s/n, 18071 Granada, Spain.

[2]Biomedical Research Center (CIBM), Biotechnology Institute, Avda del Conocimiento s/n, 18100 Armilla, Granada, Spain.

[3]Department of Soil Microbiology and Symbiotic Systems, Estación Experimental del Zaidín, Spanish National Research Council (CSIC), Profesor Albareda 1, 18008 Granada, Spain

[4]Xtrem Biotech S.L., European Business Innovation Center, Avenida de la Innovación, 1, 18016, Armilla, Granada, Spain.

[5]Department of Forestry Science, Bioremediation Laboratory, Faculty of Agricultural and Forestry Science, University of La Frontera, 01145 Temuco, Chile.

***Corresponding author:**

Inmaculada Sampedro (isampedro@ugr.es)

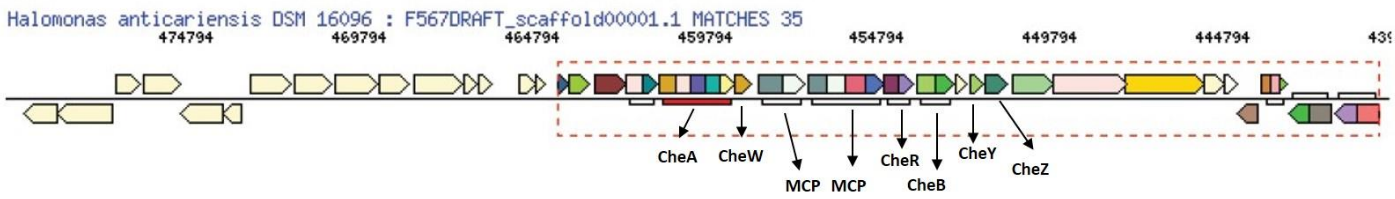

Figure S1. Chemosensory cluster identified in the draft genome sequence of *H. anticariensis* FP35^T.

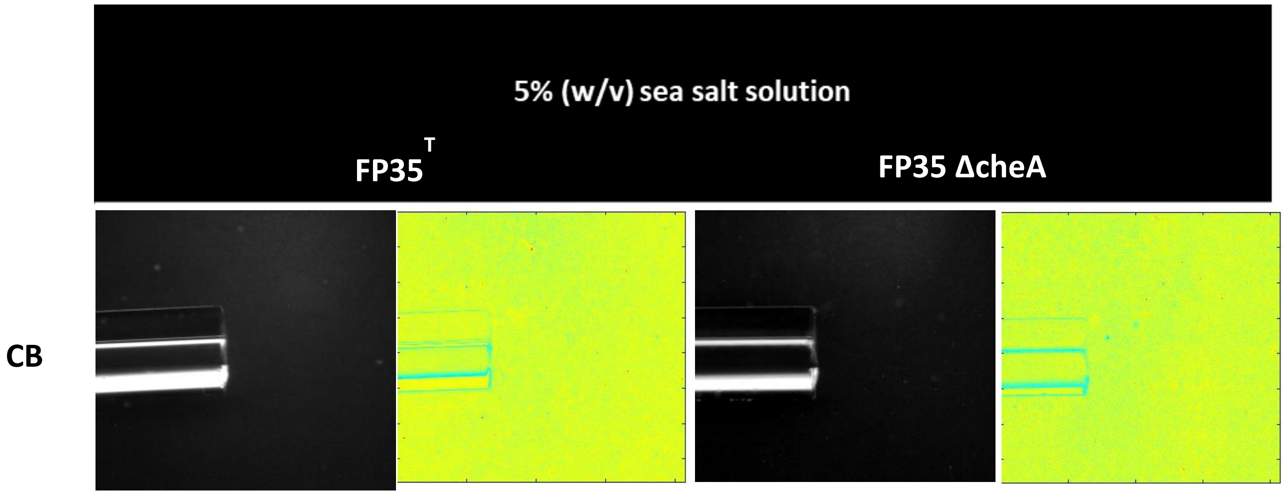

Figure S2. Qualitative capillary chemotaxis assays comparing responses of wild-type *H. anticariensis* FP35^T and the mutant strain FP35ΔcheA towards chemotaxis buffer grown at a concentration of 5% (w/v) of sea salt solution. All photographs were taken after 10 min. First and third columns: dark-field images of cells gathered at the mouth of capillaries containing chemotaxis buffer; second and fourth columns: heatmap of normalized images (Matlab R2013a).

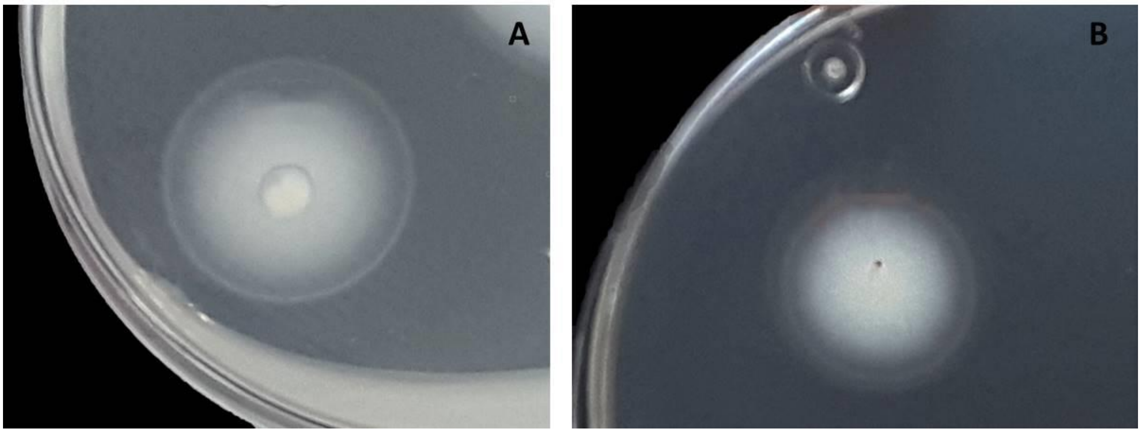

Figure S3. ***H. anticariensis*** **FP35^{T} growth in M8 plates containing 0.3% agar in absence or presence of oleanolic acid (A and B, respectively). The diameter of bacterium growth was measured using ImageJ software (A: 25.77 mm and B: 20.05 mm).**
